# *CYP26B1-*related disorder: expanding the ends of the spectrum through clinical and molecular evidence

**DOI:** 10.1007/s00439-023-02598-2

**Published:** 2023-09-27

**Authors:** Karina C. Silveira, Inara Chacon Fonseca, Connor Oborn, Parker Wengryn, Saima Ghafoor, Alexander Beke, Ema S. Dreseris, Cassandra Wong, Aline Iacovone, Carrie-Lynn Soltys, Riyana Babul-Hirji, Osvaldo Artigalas, Arthur Antolini-Tavares, Anne-Claude Gingras, Eric Campos, Denise P. Cavalcanti, Peter Kannu

**Affiliations:** 1 Department of Medical Genetics, University of Alberta, Edmonton, AB T6G 2H7 Canada; 2 Clinical Genetics, Durham Region Cancer Centre, Lakeridge Health Oshawa, Oshawa, ON L1G 2B9 Canada; 3 Genetics and Genome Biology Program, The Hospital for Sick Children, Toronto, ON M5G 0A4 Canada; 4 Lunenfeld-Tanenbaum Research Institute, Mount Sinai Hospital, Sinai Health System, Toronto, ON Canada; 5 Skeletal Dysplasia Group, Medical Genetics Area, Translational Medicine Department, FCM, University of Campinas (UNICAMP), R. Tessália V de Camargo, 126, Campinas, SP 13083-887 Brazil; 6 Division of Clinical and Metabolic Genetics, The Hospital for Sick Children, University of Toronto, Toronto, ON Canada; 7 Clinical Genetics Unit, Children’s Hospital, Grupo Hospitalar Conceicao, Porto Alegre, Brazil; 8 Department of Pathological Anatomy, University of Campinas, Campinas, 13083-888 Brazil; 9 Department of Molecular Genetics, University of Toronto, Toronto, ON M5S 1A8 Canada

## Abstract

CYP26B1 metabolizes retinoic acid in the developing embryo to regulate its levels. A limited number of individuals with pathogenic variants in *CYP26B1* have been documented with a varied phenotypic spectrum, spanning from a severe manifestation involving skull anomalies, craniosynostosis, encephalocele, radio-humeral fusion, oligodactyly, and a narrow thorax, to a milder presentation characterized by craniosynostosis, restricted radio-humeral joint mobility, hearing loss, and intellectual disability. Here, we report two families with *CYP26B1*-related phenotypes and describe the data obtained from functional studies of the variants. Exome and Sanger sequencing were used for variant identification in family 1 and family 2, respectively. Family 1 reflects a mild phenotype, which includes craniofacial dysmorphism with brachycephaly (without craniosynostosis), arachnodactyly, reduced radioulnar joint movement, conductive hearing loss, learning disability—and compound heterozygous *CYP26B1* variants: (p.[(Pro118Leu)];[(Arg234Gln)]) were found. In family 2, a stillborn fetus presented a lethal phenotype with spina bifida occulta, hydrocephalus, poor skeletal mineralization, synostosis, limb defects, and a synonymous homozygous variant in *CYP26B1*: c.1083C > A. A minigene assay revealed that the synonymous variant created a new splice site, removing part of exon 5 (p.Val361_Asp382del). Enzymatic activity was assessed using a luciferase assay, demonstrating a notable reduction in exogenous retinoic acid metabolism for the variant p.Val361_Asp382del. (~ 3.5 × decrease compared to wild-type); comparatively, the variants p.(Pro118Leu) and p.(Arg234Gln) demonstrated a partial loss of metabolism (1.7× and 2.3× reduction, respectively). A proximity-dependent biotin identification assay reaffirmed previously reported ER-resident protein interactions. Additional work into these interactions is critical to determine if CYP26B1 is involved with other biological events on the ER. Immunofluorescence assay suggests that mutant CYP26B1 is still localized in the endoplasmic reticulum. These results indicate that novel pathogenic variants in *CYP26B1* result in varying levels of enzymatic activity that impact retinoic acid metabolism and relate to the distinct phenotypes observed.

**Supplementary Information:**

The online version contains supplementary material available at 10.1007/s00439-023-02598-2.

## Introduction

The temporospatial control of retinoic acid (RA) gradients is crucial during early embryogenesis (Hernandez et al. 2007; Stoney et al. 2016). CYP26 genes, including CYP26A1, -B1, and -C1, are responsible for regulating RA levels by breaking down all-trans-RA. These genes are part of the cytochrome P450 family and encode heme-containing enzymes found in the endoplasmic reticulum (ER) membrane (Cunningham and Duester 2015). RA excess, either due to increased dietary intake of vitamin A, or reduced catabolism by CYP26 enzymes, is associated with craniofacial malformations and limb defects in animal models (Hendrickx and Hummler 1992; Laue et al. 2008). *CYP26B1* (located at 2p13.2, genomic coordinates: 72,129,238–72,147,862, GRCh38/hg38) encodes for the CYP26B1 enzyme which is important for the development and differentiation of the central nervous system (especially the hindbrain) and skeletal development, where it regulates patterning, bone ossification, and the establishment of specific synovial joints (Hernandez et al. 2007; Cunningham and Duester 2015; Stoney et al. 2016).

Pathogenic variants affecting the CYP26B1 enzyme cause a range of phenotypes affecting the skeletal, dental, nervous and visual systems (Laue et al. 2011; Morton et al. 2016; Grand et al. 2021). Thus far, three disease-causing homozygous missense variants in *CYP26B1* have been described (Laue et al. 2011; Morton et al. 2016). The p.(Arg363Leu) variant results in a lethal phenotype with defective calvaria, occipital encephalocele, radio-humeral synostosis, advanced osseous maturation, arachnodactyly, terminal phalangeal aplasia of the thumbs and great toes, bent femurs and a narrow thorax (Laue et al. 2011). The p.(Ser146Pro) variant results in a severe but survivable phenotype characterized by craniosynostosis, a large sagittal skull defect, and limited elbow extension with arachnodactyly (Laue et al. 2011). The third homozygous variant (p.Gly435Ser) was identified in an individual with a mild phenotype presenting with multiple craniosynostosis, radio-humeral synostosis, bilateral hearing loss, and mild intellectual disability (Morton et al. 2016). All individuals identified with *CYP26B1* compound heterozygous missense variants (p.[(Arg363His)];[(Arg397Gln)], p.[(Arg234Gln);[(Gly126Cys)]) have mild to intermediate skeletal malformations including craniosynostosis, delayed skull ossification, cranial lacunae, midface retrusion, a narrow upper thorax, scoliosis, limited elbow extension, radio-ulnar synostosis, hand deformities including 1st digit oligodactyly, short distal thumb phalanges, camptodactyly, gracile bones, dysmorphic features including small cupped ears, a prominent nose, and mild cognitive impairments associated with conductive hearing loss (Grand et al. 2021).

Here, we describe two unrelated families with distinct phenotypes due to novel pathogenic variants in *CYP26B1*. Compound heterozygous *CYP26B1* variants (p.[(Pro118Leu)];[(Arg234Gln)]) in family 1 result in a mild phenotype including a Marfanoid habitus, bony synostosis, learning disability, and abnormal cranial shape without craniosynostosis. In contrast, a homozygous variant (p.[Val361_Asp382del];[Val361_Asp382del]) in family 2 leads to a lethal phenotype marked by decreased skeletal mineralization, and defects in the skull, spine, brain, and limbs. Our study aimed to describe the skeletal phenotypes associated with these rare variants and uncover their molecular mechanisms, thus expanding our understanding of the range of defects connected to RA catabolism.

## Materials and methods

### Chromosome analysis and genotyping

The variants reported here were deposited in the Global Variome shared LOVD (https://www.lovd.nl/) under IDs #0000870065, #0000870066, and #0000870081 (reference sequence: NM_019885.4, GRCh38/hg38). Primer sequences are available in the online supplemental Table S1.

*Family 1.* Chromosome microarray analysis and craniosynostosis gene panel testing (*FGFR3*; *TWIST1; FGFR1*, p.Pro252Arg; *FGFR2*, IIIa and IIIc) were performed. The phenotype was reminiscent of the Shprintzen–Goldberg Syndrome (OMIM 182212) for which genetic testing was unavailable at the time. Quadruplet whole exome sequencing (WES) was subsequently undertaken (GeneDx: https://www.genedx.com/) using DNA samples from both parents and both affected individuals.

*Family 2*. Exons and intron–exon boundaries of *CYP26B1* were amplified from parental genomic DNA (peripheral blood). The amplicons were sequenced by Sanger sequencing (SS) with the BigDye^®^ Terminator method following the manufacturer’s instructions. Exon 5 was also investigated by SS of proposita’s DNA sample from FFPE (Formalin-fixed, paraffin-embedded) tissue.

### In silico analysis and frequency in populational databases

Variant pathogenicity was accessed using the ACMG classification, Mutation Taster (http://www.mutationtaster.org/), and Franklin (https://franklin.genoox.com). Variant frequency was verified in Exome Variant Server (https://evs.gs.washington.edu/EVS/), gnomAD (https://gnomad.broadinstitute.org/), ABraOM (http://abraom.ib.usp.br/), Human Gene Mutation Database (http://www.hgmd.cf.ac.uk/ac/index.php), and ClinVar (https://www.ncbi.nlm.nih.gov/clinvar/). Prediction of splicing defects was performed using the following tools: *BDGP* (https://www.fruitfly.org/seq_tools/splice.html), *Human Splicing Finde*r (http://umd.be/Redirect.html), SpliceAI (https://spliceailookup.broadinstitute.org/). The consensus region of *CYP26B1* donor splice sites was analyzed to verify the conservation of the region affected by the c.1083C > A variant (online supplemental Fig. S1).

### *CYP26B1* minigene splicing assay

The effect of the c.1083C > A variant on splicing was explored using a minigene assay, following a previously described protocol (Desviat et al. 2012). Briefly, a PCR product including exon 5 (285 bp) and exon 6 (393 bp) plus 143, 403, and 73 nucleotides from intron 4, intron 5, and 3’UTR was amplified using DNA from peripheral blood of a healthy control. The PCR product (insert) was cloned into the *pCR*^*™*^*2.1-TOPO*^®^ vector (TOPO^®^ TA Cloning^®^ Kit, Invitrogen^™^). The insert was then excised (*BamHI* and *XbaI* restriction enzymes) and ligated into the Exontrap vector *pET01* (MoBiTec GmbH). Once the wild-type (WT) minigene was constructed, the c.1083C > A variant was introduced by site-directed mutagenesis using the QuikChange XL Site-Directed Mutagenesis Kit (Agilent Technologies) following the manufacturer’s instructions. WT and mutant minigene sequences were confirmed by SS (Molecular Biology Service Unit, MBSU, University of Alberta).

*Cell culture and transfection.* 1.5 × 10^4^ COS-7 cells (African green monkey kidney fibroblast-like cell line, courtesy of Randal Nelson—University of Alberta) were plated in 24-well plates and grown to 90% confluency in 0.5 ml of medium (DMEM, high glucose, pyruvate; 10% fetal bovine serum; 1% Penicillin–Streptomycin 5000 U/mL). Then, 250 ng of WT and mutant minigenes were transfected in triplicate into COS-7 cells using Lipofectamine 2000 (Thermo Fisher Scientific) in Gibco Opti-MEM^™^ I Reduced Serum Medium (Thermo Fisher Scientific). After 3 days, total RNA was isolated using TRIzol/chloroform (Invitrogen, Carlsbad, CA, USA) according to the manufacturer’s instructions.

*Transcript amplification.* Reverse transcription was carried out with 1 µg of RNA and SuperScript™ III First-Strand Synthesis System (Invitrogen), using a specific primer to the 3′ native exon in the pET01 vector. Samples were incubated at 50 °C for 1 h, followed by 15 min at 70 °C. Transcripts were amplified with PCR SuperMix (Life Technologies) using 1 µl of cDNA (50 ng/µl) and the *CYP26B1* primers PCR consisted of a denaturation step at 94 °C for 5 min, a first annealing step of 20 cycles 94 °C-30 s, 71 °C-30 s (gradually reducing 0.5 °C/cycle) and 72 °C-40 s followed by a second annealing step of 17 cycles of 94 °C-30 s, 65 °C-30 s and 72 °C-40 s, and a final extension step at 72 °C for 10 min. The resulting amplified fragments were visualized on a 1.2% agarose gel and verified by SS.

### Generation of expression plasmids

A human *CYP26B1* construct in *pcDNA3.1* was tagged at the C-terminus with 3XFLAG obtained from GenScript (#OHu20816, https://www.genscript.com/). Site-directed mutagenesis was performed on the *CYP26B1* plasmids to insert the variants identified in families 1 and 2 as well as the previously reported variant c.1088G > T (Laue et al. 2011).

### Western blot

Western blots were conducted in two different cell lines in triplicate and repeated three different times. 2.5 × 10^5^ NIH/3T3 cells (ATCC, CRL-1658) were plated on 6-well plates for 24 h and then transiently transfected with each of the constructs using Lipofectamine^™^ LTX Reagent (Invitrogen^™^, #15,338,100), following the manufacturer’s instructions. In addition, 2.5 × 10^5^ HEK293T/17 cells (ATCC, CRL-11268) were plated on 6-well plates for 24 h and then transiently transfected with each of the constructs using Lipofectamine 2000 (ThermoFisher, #11,668,019), following the manufacturer’s instructions. After 48 h, cells were scraped with 1% NP40 lysis buffer (20 mM Tris pH 7.4, 5 mM EDTA, 10 mM Na_4_P_2_O_7_, 100 mM NaF), phosphatase inhibitor (Millipore, 524,628), a protease inhibitor (Sigma, P8340), and sodium orthovanadate, sheared with a 26½ gauge needle, and cleared by centrifugation at 1200×G for 30 min. Each sample was quantified via a BCA assay. Concentration-adjusted samples were run on an SDS gel, transferred to a nitrocellulose membrane, and blocked in 5% Skim-Milk solution. Blots were rotated overnight with the primary antibody at 4 °C (Mouse anti-FLAG-M2 [Sigma F3111], 1:1000; Mouse anti-β-Actin [ABCAM AB6276, AC-15], 1:1000). The next day, blots were incubated using a HRP-linked Goat anti-Mouse IgG (Cell Signalling 7076S, 1:1000) secondary antibody for 1 h. Chemiluminescence was achieved through the Western Lighting ECL Plus kit (Perkin Elmer) on ChemiDoc^™^ MP (Imaging System, Bio-Rad).

### CYP26B1 dual-luciferase reporter assay (DLR)

To investigate CYP26B1 functional activity, a DLR assay was performed as previously described (Laue et al. 2011). Briefly, HEK293T/17 cells (ATCC, CRL-11268) were seeded at 1.5 × 10^4^ cells/well in a 24-well plate. Cells were co-transfected after 24 h with a RARE reporter plasmid (*pGL3-RARE-luciferase*, AddGene #13,458), control plasmid containing Renilla (pgL3_pRL_TK), and a *CYP26B*1 construct (WT *CYP26B1*, or *CYP26B1* with c.1083C > A, c.353C > T, c.701G > A, c.1088G > T (Laue et al. 2011)). After 48 h, treatment with retinoic acid/complete medium at 1 µM diluted in DMSO was administered. The cells were incubated for 24 h and then assayed with a Dual-Luciferase Reporter Assay System (Promega, #E1910) and the read-out was measured on a Turner Designs TD-20/20 Luminometer. The experiment was performed in triplicate and repeated in five independent experiments.

### BioID

The proximity-dependent biotin identification of associating proteins (BioID) analysis for CYP26B1 was undertaken as previously described with minor changes (Scott et al. 2021). CYP26B1 cDNA was cloned into pDEST–pcDNA–BioID2–FLAG N-term (courtesy of Dr. Anne-Claude Gingras) to express proteins tagged with the BioID2 enzyme. GFP cDNA was also introduced into the plasmid and was used as a negative control for the BioID experiment. Inducible isogenic HEK293 Flp-In T-REx (ThermoFisher Cat# R78007) lines expressing GFP or WT or mutant CYP26B1 were generated. Proteins were biotinylated for 8 h and cells were harvested. Five or more 150 mm plates of 80% confluent cells were harvested for each biological replicate. Samples were processed and analyzed by mass spectrometry as previously described in with the following modifications (Scott et al. 2021): 1.8 ml of cleared lysate was incubated with the streptavidin Sepharose beads and 1/4 of the sample was acquired on a TripleTOF6600 (AB Sciex, Concord, Ontario, Canada) using a 90 min gradient from 2% acetonitrile to 35% acetonitrile with 0.1% formic acid. Mass spectrometry acquisition in data-dependent mode consisted of one 250 ms MS1 scan from 400 to 1800 Da, followed by ten 100 ms MS2 scans from 100 to 1800 Da in high sensitivity mode. Only ions with 2–5 charge state and 300cps or higher were selected for MS2, with a dynamic exclusion time of 7 s.

Two independent replicates were used for the BioID analysis. Non-specific associations with the GFP-BioID2 fusion protein were filtered out using the SAINT-express probabilistic scoring tool, as previously described (Teo et al. 2014). Protein associations were considered if they had a Bayesian false discovery rate (BFDR) ≤ 1%. Further analyses considered proteins with an average spectral count of at least 10 in with the WT or mutant CYP26B1 BioID. Gene ontology terms were identified using g:Profiler (Raudvere et al. 2019). Data were visualized using ProHits-Viz, with data normalized to the total abundance (Knight et al. 2017). Mass spectrometry data for the BioID has been deposited to the MassIVE repository (https://massive.ucsd.edu/ProteoSAFe/static/massive.jsp) and assigned the accession number MSV000091800 (ftp://massive.ucsd.edu/MSV000091800). The ProteomeXchange accession is PXD041830.

### Protein modelling

The 3D structure of retinoic acid-bound cyanobacterial CYP120A1 (highest sequence similarity with CYP26B1) was retrieved from the RCSB Protein Data Bank (PDB ID 2VE3) (Kühnel et al. 2008). The FASTA sequence of human CYP26B1 was retrieved from Uniprot (Q9NR63). WT and two individual specific CYP26B1 protein models (modified FASTA files for p.Val361_Asp382del and for p.Pro118Leu with p.Arg234Gln) were generated ab initio using the AlphaFold2 ColabFold notebook with default settings and ranked by pLDDT (Jumper et al. 2021; Mirdita et al. 2021). The highest overall ranked model PDB files and 2VE3 were used for further analysis and loaded into PyMOL v2.0 (The PyMOL Molecular Graphics System, Version 1.2r3pre, Schrödinger, LLC). The structural assessment was done using the ExPasy Swiss-Model Structural Assessment tool for the predicted WT model.

### Histopathology and immunofluorescence assay

*H&E staining.* Formalin-Fixed Paraffin-Embedded (FFPE) fetal growth plate sections from family 2 were cut at a thickness of 4 µm and used for hematoxylin and eosin (H&E) staining.

*Immunofluorescence assay: *NIH/3T3 cells (ATCC, CRL-1658) were seeded at 1 × 10^5^ on glass coverslips coated with poly-l-lysine in 6-well plates. Cells were transfected with either p.Val361_Asp382del, p.Pro118Leu, p.Arg234Gln, and p.Arg363Leu *CYP26B1* constructs using Lipofectamine^™^ LTX Reagent (Invitrogen^™^,, #15,338,100), following the manufacturer’s instructions. After 24 h, media was removed, cells were fixed with 4% paraformaldehyde at room temperature for 15 min, then permeabilized with 0.1% Triton for 5 min at room temperature and blocked with 3% BSA for 1 h, washed with PBS 3X and incubated with the anti-FLAG (1:200, Monoclonal ANTI-FLAG^®^ M2 antibody, mouse, #F1804, Sigma-Aldrich) and the anti-Calnexin (1:200, Calnexin Polyclonal antibody, rabbit, #10,427-2-AP, Proteintech) primary antibodies for 2 h. Cells were washed with PBS 3X and incubated with secondary antibodies for 1 h (1:500, Alexa Fluor 594 goat anti-mouse, #A11005 and Alexa Fluor 488 goat anti-rabbit antibody, #A11034, Invitrogen). Cells were washed again with PBS 3X, stained with Hoechst 33,342 at 1 ug/ml (Molecular Probes, #H-3570,) for 15 min, washed with PBS 3X, and mounted on slides with ProLong^™^ Gold Antifade Mountant (Invitrogen, #P36930,). Fluorescence microscopy was done at 60× in the Spinning Disk Confocal Microscope (Quorum Technologies) at the Cell Imaging Core at the University of Alberta.

### Statistical analysis

Statistical analyses were performed in Prism (GraphPad) using the one-way analysis of variance (ANOVA) with the corrected Bonferroni post hoc test for multiple comparisons. The significance level was set to 0.05. All experiments were performed in triplicate in three independent experiments, except the DRL which was performed five times.

## Results

### Patients’ description

**Fig. 1:**
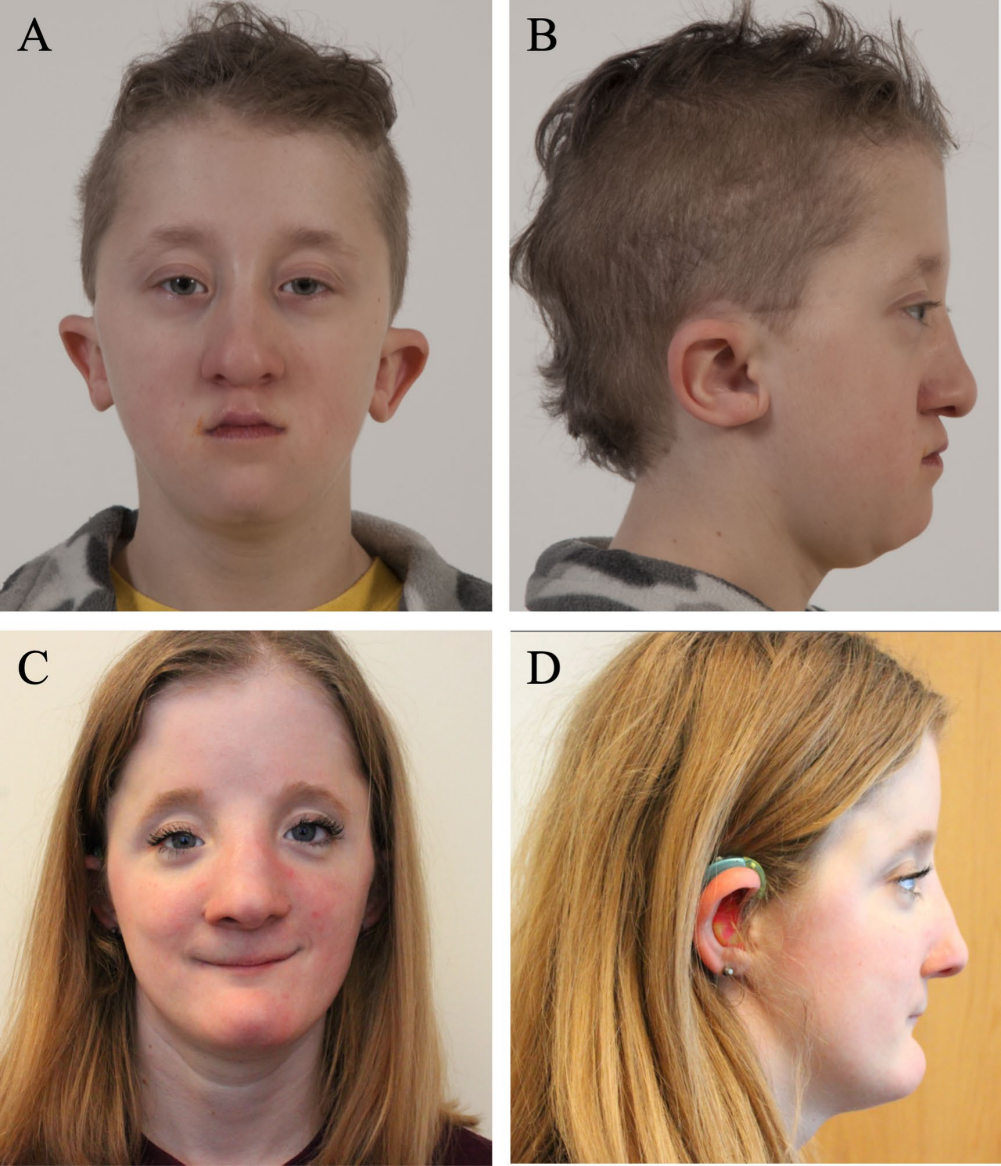
Clinical features of siblings from family 1. **A**, **B** Facial characteristics of the proband (11 years) and his more severely affected sister (**C**, **D** 15 years). Note turribrachycephaly with a very high forehead and triangular face, interrupted eyebrows, shallow orbits, and small-looking palpebral fissures

*Family 1*. The proband (Fig. 1A, B, online supplemental Fig. S2 and S3) was first seen at 17 months of age. He was born early at 35 week gestation because of pre-eclampsia. An abnormal skull shape was previously identified on fetal ultrasound and craniofacial dysmorphisms were noted at birth. In addition, he had a small and narrow oropharynx and ear canals, small nasal airways, and a right diaphragm paralysis. An ophthalmic examination revealed normal optic nerves but no other deformities except for epiphora and hyperopia. He subsequently developed chronic rhinitis, severe conductive hearing loss (requiring hearing aids), eczema, chronic constipation, and joint stiffness affecting the fingers/toes, elbows, and ankles. He suffers episodic headaches and has experienced several seizure-like episodes. He has mild developmental delay and learning disabilities requiring an individualized education plan, especially for reading and math.

His older sister (Fig. 1C, D, online supplemental Figs. S2 and S3) has a similar phenotype. Her antenatal period was unremarkable except for the identification of an abnormal cranial shape on fetal ultrasound. Despite third-trimester pre-eclampsia, the pregnancy continued to term and she was born via a normal vaginal delivery. Her medical history was significant for recurrent otitis media due to narrow external ear canals and conductive hearing loss requiring hearing aids. She also had nasal polyps, a nasal septal deviation and a nasolacrimal duct obstruction that improved with time. She complained of recurrent/voluntary shoulder dislocation. Her ophthalmologic examination was normal except for mild peripapillary atrophy which was not clinically relevant. She suffered from mild asthma, eczema, and migraine-like episodic headaches. She was also diagnosed with mild developmental delays and learning disabilities, especially in reading/comprehension and math. Craniofacial reconstructive surgery was undertaken at age 10.

The siblings' physical examination revealed strikingly similar features summarized in supplemental Table S2. Their head circumference and upper-to-lower body ratio were normal. Other common features included turribrachycephaly with a very high forehead and triangular face, shallow orbits and small-looking palpebral fissures, a prominent nose with a high bridge and bulbous tip, a small philtrum and mouth, a high palatal arch and crowded teeth, a flat facial profile due to malar hypoplasia, pointy chins and prominent low set and posteriorly rotated ears. Only the female sibling presented ocular hypertelorism. The proband had pectus excavatum, a short fourth metatarsal, and overriding of his fourth left toe. Both siblings displayed a Marfanoid habitus with slim limbs, arachnodactyly, and a drumstick appearance to the distal phalanges and long toes. Bony protrusion at the ulnar area of the wrist (palmar side) and restricted pronation/supination movements were identified in both. Their parents were non-consanguineous, of Caucasian descent and had another healthy child together. In addition, each parent also had a healthy child from a previous relationship.

Head computerized tomography (CT) in the proband was performed a few months after birth and ruled out craniosynostosis but showed wide orbital fissures, thought to be related to the partial absence of the medial portions of the greater sphenoid wing, and maxillary hypoplasia. There was prominence of the extracerebral space and mild ventriculomegaly. A brain MRI at age 11 years showed nonspecific multiple foci of white matter hyperintensities distributed in the left parietal and bifrontal areas and left periventricular gliosis. His skeletal survey at 13 years showed a flattened appearance of the frontal and midface bones and a pointy mandibula. A short left fourth metatarsal bone and fusion of the left second metatarsal and medial cuneiform were identified. Both piriformes appear elongated, extending peripherally and projecting over the hamate. His bone age was consistent with his chronological age.

His sister’s head CT excluded craniosynostosis but identified enlarged subarachnoid spaces with enlarged ventricles. She also had a partial absence of the greater sphenoid wings bilaterally resulting in an enlarged appearance of the orbital fissures and maxillary hypoplasia. A skeletal survey done at 15 years of age revealed subtle mid-thoracic scoliosis and a slightly exaggerated lumbar lordosis. There was no evidence of elbow joint fusions, although there was a suspicion of a bony bar between the right radial head and proximal ulna. She had elongated metatarsals, phalanges, and metacarpals. Bone mineralization was preserved with a bone age of 17y (chronological age 15 y 5 m), still within 2SD and considered appropriate skeletal maturation.

**Fig. 2:**
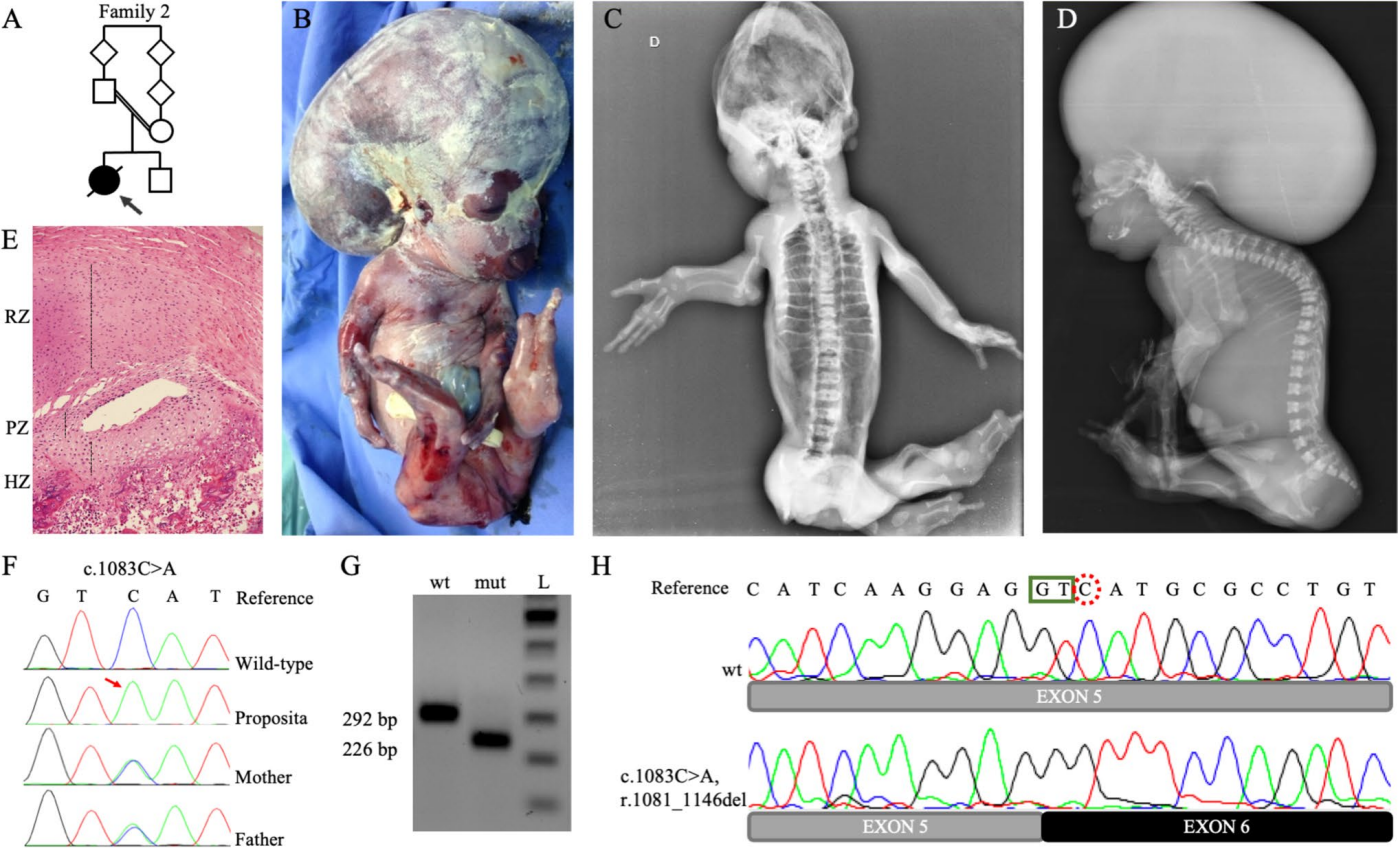
Clinical features and genetic investigation of proposita from family 2. **A** Family pedigree. **B** Lateral view of the fetus. Note dolichocephaly, very small ears, lingual protrusion, short limbs with oligodactyly—the left foot presents an absence of hallux and a single middle toe with an appearance of arachnodactyly. **C**, **D** Babygram presents a skeleton poorly mineralized, the vault not calcified, absence of clavicles, generalized spina bifida occulta, severe thoracic kyphosis with tall vertebrae (better seen in the lateral view), short and thin ribs, hypoplasia of scapulae, short humerus with shaft fracture at right, synostosis of the elbow joint, carpal and tarsal ossification nucleus, four metacarpal bones, thumb with one phalanx, the next two fingers with two phalanges and the last with only one phalanx, hypoplastic femur, bowing of tibiae and fibulae are bowing, presence of three metatarsals and only a single toe with a single phalanx. **E** Growth plate of the fetus showing the resting zone (RZ) with slight hypercellularity of the chondrocytes, a short proliferative zone (PZ) with small cells with poor cytoplasm, and hypertrophic zone (HZ), also short, and few cells more widely spaced than expected. **F** Electropherogram of exon 5 displaying the variant c.1083C > A in homozygosity in the proposita (red arrow) and in heterozygosity in both parents. **G** RT-PCR analysis of the minigene assay product comparing the wild-type (wt) and the mutant (mut) plasmid. Note the difference in sizes, showing the deletion of 66 amino acids caused by the splicing defect. **H** Electropherogram of the RT-PCR product showing the exact breakpoint of the splicing. *bp* base pair; *L* 100 bp ladder

*Family 2.* The proposita in family 2 (Fig. 2B–D) was the first pregnancy of a healthy and consanguineous Brazilian couple (*F* = 1/32, Fig. 2A). There was no family history of congenital defects. The parents are of a Caucasian background and originate from a southern Brazilian town with a Polish settlement. A prenatal ultrasound at 31 + 5 weeks identified a single fetus with associated polyhydramnios, voluminous hydrocephalus (cephalic circumference = 35.1 cm [*z*-score + 4.92]), an elliptical skull shape, agenesis of the corpus callosum and absence of posterior fossa elements. The abdominal circumference was 21.9 cm (*z*-score – 3.47), and the long bones were short (below p 2.5). Severe kyphosis, a narrow thorax, and pulmonary hypoplasia were also observed. Mineralization was decreased, especially in the long bones. The following skeletal defects were observed: bowing of humerus, radius, and ulnae, absence of the femora, shortening of the tibiae, and abnormally shaped hands and feet with a lack of some fingers and toes. The phenotype was consistent with a lethal skeletal disorder; the sonographer raised two hypotheses—hypophosphatasia (OMIM 241500) and osteogenesis imperfecta (OMIM 166210). The fetus, a stillborn female, was born at 33 weeks by cesarian section. Growth birth parameters were: birth weight 539 g (*z*-score – 0.92); birth length 41.3 cm (*z*-score – 0.51); and occipital frontal circumference 41.0 cm (*z*-score + 7.57). A review of clinical photographs showed a large head with dolichocephaly, a very small and low set right ear, mild ocular protrusion, lingual protrusion, a short neck, thoracic kyphosis, short limbs and four finger oligodactyly, including the right thumb, and feet with a single median toe.

Radiographs revealed generalized osteopenia and absent skull vault ossification. The following findings were also observed: absent clavicles, hypoplastic scapulae, abnormal vertebral pedicle morphology in the anterior–posterior view compatible with generalized spina bifida occulta, severe thoracic kyphosis and short and thin ribs. Lateral spinal views indicated the appearance of tall vertebrae. Both humeri were short and there was a right midshaft humeral fracture. In addition, elbow joint synostosis and a large carpal ossification nucleus (probably synostosis of capitate-hamate) were also observed. The right hand presented only four metacarpal bones and the left hand was not evaluated; the thumb showed one phalanx, the next two fingers had two phalanges, and the last one had only one phalanx. Both femora were severely hypoplastic, the tibiae and fibulae were bowed, two tarsal ossification centres were noted, and there were three metatarsals and a single phalanx in the middle toe. The pelvis could not be seen in the available radiographs (Fig. 2C, D).

The autopsy revealed hydrocephalus and cerebral atrophy due to lateral ventricle dilatation and compression of the parenchyma, deformity of the cranial base bones; an apparently normal spinal cord despite complete spina bifida occulta (absence of the posterior process of the vertebrae from C1 to S1); and hypoplasia of the lower pulmonary lobes, without other internal anomalies. Detailed clinical findings are described in the online supplemental Table S3. Four years after the proposita’s birth, the parents had a healthy boy.

Femoral growth plate histology revealed disorganization of the resting zone and hypercellularity; the proliferative and hypertrophic zones were short with small cells and reduced cytoplasm in the proliferative zone and fewer more widely spaced cells in the hypertrophic zone. Osteocytes and osteoblasts were observed in the trabecular zone (Fig. 2E).

### Genetic testing

*Family 1*. Chromosomal microarray analysis and targeted single gene variant testing (*FGFR1* (p.P252R), *FGFR2* (IIIa and IIIc), *FGFR3* and *TWIST1*) was uninformative. Later, quadruplet (parents and affected siblings) whole exome sequencing (WES) was undertaken and revealed two compound heterozygous variants of unknown significance (VUS) *in trans* in the *CYP26B1* gene (c.353C > T; p.(Pro118Leu) and c.701G > A; p.(Arg234Gln)) in both siblings. The first variant, c.353C > T in exon 2 was paternally inherited and absent in large population cohorts. The second variant c.701G > A in exon 3, was maternally inherited and has been observed with an allele frequency of 0.00004631 (Popmax filtering allele frequency = 0.0003353, gnomAD—https://gnomad.broadinstitute.org/).

*Family 2.* Initial SS analysis revealed that the parents were heterozygous for the c.1083C > A variant (Fig. 2F) in exon 5. Later, SS of exon 5 of the proposita’s DNA confirmed the c.1083C > A variant in homozygosity. This variant is absent in gnomAD, EVS, ABraOM, HGMD, and ClinVar databases. The variant encoded a synonymous change (p.(Val361 =)) but further *in-silico* analysis suggested the creation of a new cryptic donor splice site, prompting the hypothesis that this variant led to a partial deletion of exon 5.

### Functional analysis of the *CYP26B1* variants

Minigene analysis revealed a near-exclusive expression of an abnormally spliced transcript (226 bp) for the c.1083C > A variant (Fig. 2G). Sequencing of the amplicon confirmed a 66-nucleotide exon 5 deletion. (Fig. 2H). In comparison, the WT minigene only expressed the normally spliced exon 5 isoform (292 bp, Fig. 2G). Therefore, the variant was re-classified as “pathogenic” (PVS1_strong, PS3, PM2, and PP4) according to ACMG guidelines and Clingen recommendations (Richards et al. 2015; Abou Tayoun et al. 2018).

**Fig. 3:**
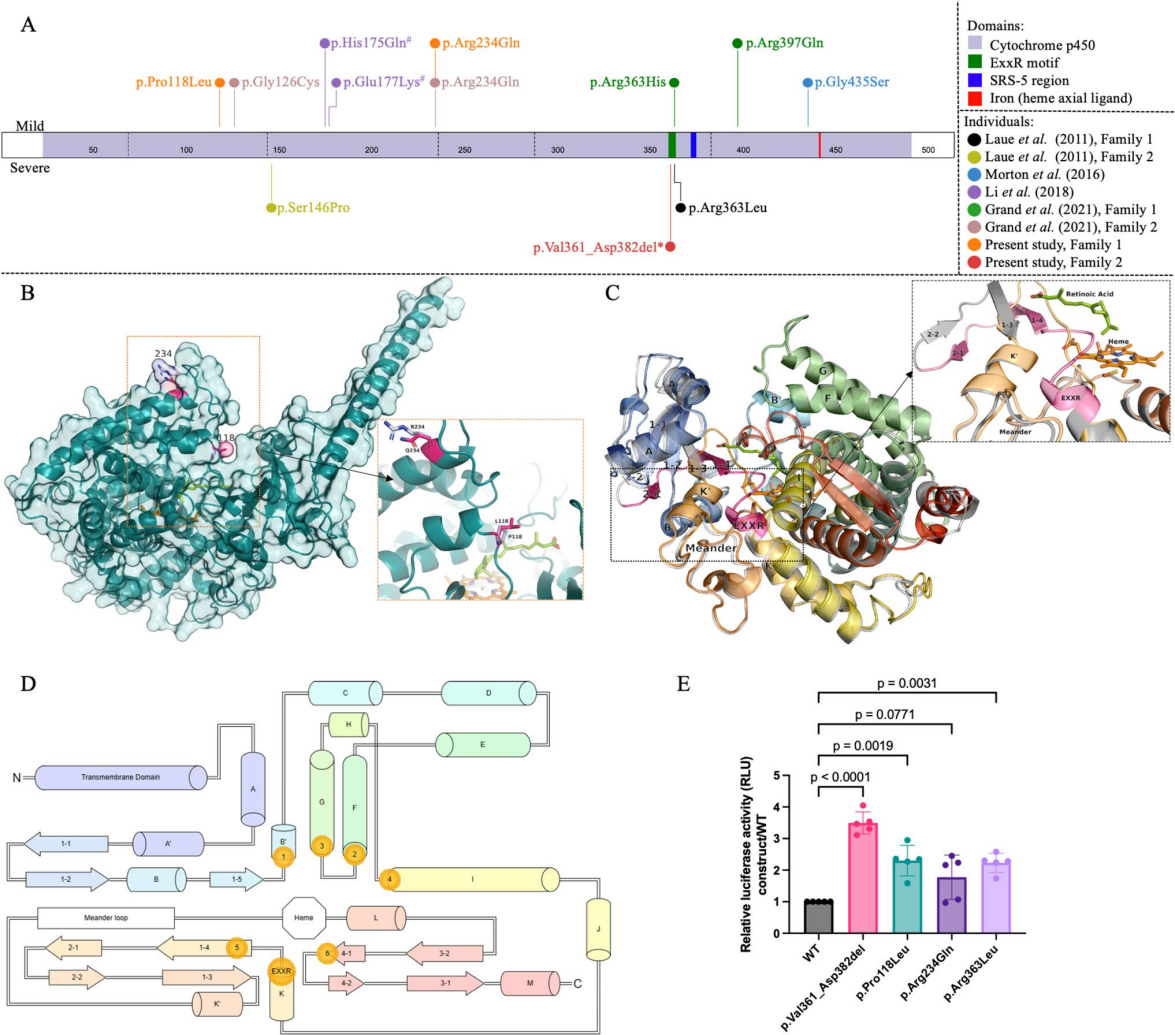
**A** Described variants along *CYP26B1*. The variants on the top are found in patients presenting mild phenotypes and those on the bottom represent variants in patients with a severe or lethal phenotype. Individuals are referenced on the right and variants are colour-coded by individual (two variants with the same colour represent compound heterozygosity). The figure was adapted from the ProteinPaint website (https://proteinpaint.stjude.org/). **B** Structural consequences of p.Pro118Leu and p.Arg234Gln substitutions. Missense variant substitutions are labelled in pink with the wild-type (WT) in blue, retinoic acid and heme are present in the interior cavity coloured lime and orange, respectively. **C** Predicted p.Val361_Asp382del structure superimposed on WT CYP26B1. The pink region represents the WT peptide sequence that is deleted resulting in the chromatic predicted structure; this region extends from the ExxR motif through *β* 2–2. **D** CYP26B1 as a topological schematic. All secondary structures are annotated chromatically from N to C termini, alpha helices are represented as tubes and beta strands as directional arrows. General locations of the cytochrome p450 conserved substrate recognition sites and ExxR motif are labelled with gold disks. The meander loop is defined as the region between αK’ and the heme binding site shown by a white octagon. Adapted from (36). **E** Luciferase assay results showed the normalized fold change in activity (constructs/WT) when cells were treated with RA. *N* = 5 independent experiments. Statistical analysis was performed using ANOVA followed by a corrected Bonferroni post hoc test. *WT* wild-type plasmid, *NTC* non-transfected cells, ^#^ Individual also has pathogenic variants in the gene *NAGLU* (14)*.* All pathogenic variants described here in the *CYP26B1* gene are numbered according to the reference sequence NM_019885.4 (reference genome GRCh38, hg 18)

Ectopic expression of the p.Val361_Asp382del protein caused an accumulation of intracellular RA (~ 3.5-fold increase in luciferase activity), confirming a notable loss in the ability to metabolize RA. The difference in the enzymatic function was significant between p.Val361_Asp382del and the other tested variants. The other two variants, p.(Pro118Leu) and p.(Arg234Gln), also caused a partial loss of CYP26B1 enzymatic activity, with 2.3- and 1.7-fold increases in luciferase activity, respectively (Fig. 3E). The enzymatic function did not differ significantly between the p.Arg234Gln variant and the other constructs. Finally, the p.Arg363Leu variant showed a 2.1-fold increase compared to the WT. Western blotting of transiently transfected HEK293T/17 and NIH/3T3 cells confirmed similar expression levels for the various CYP26B1 constructs (online supplemental Fig. S4).

### Protein modelling

Protein modelling revealed that both WT and missense variant structures show a small amount of variance in canonical cavity formation. However, a large change in the interior is visible with the p.Val361_Asp382del variant (Fig. 3B, C). p.(Pro118Leu) and p.(Arg234Gln) variants were present right before αB’ and at the start of αG on the structure, respectively. The p.(Pro118Leu) variant resulted in a non-conservative substitution right at an opening used by RA to enter and bind to the enzyme active site. Since leucine is bulky in comparison, we hypothesize that the mutant enzyme is less efficient at binding RA. Conversely, the p.(Arg234Gln) variant is present at one of the putative membrane contact points for this protein and swaps a positively charged membrane contact for an uncharged polar contact. The p.Val361_Asp382del variant involves the end component of αK that contains the ExxR motif and disrupts the first and second beta sheets creating a noncanonical association between β 1–3 and residues near β 2–2 (Fig. 3C).

### Proteomic analysis of WT and variant CYP26B1 proteins

**Fig. 4:**
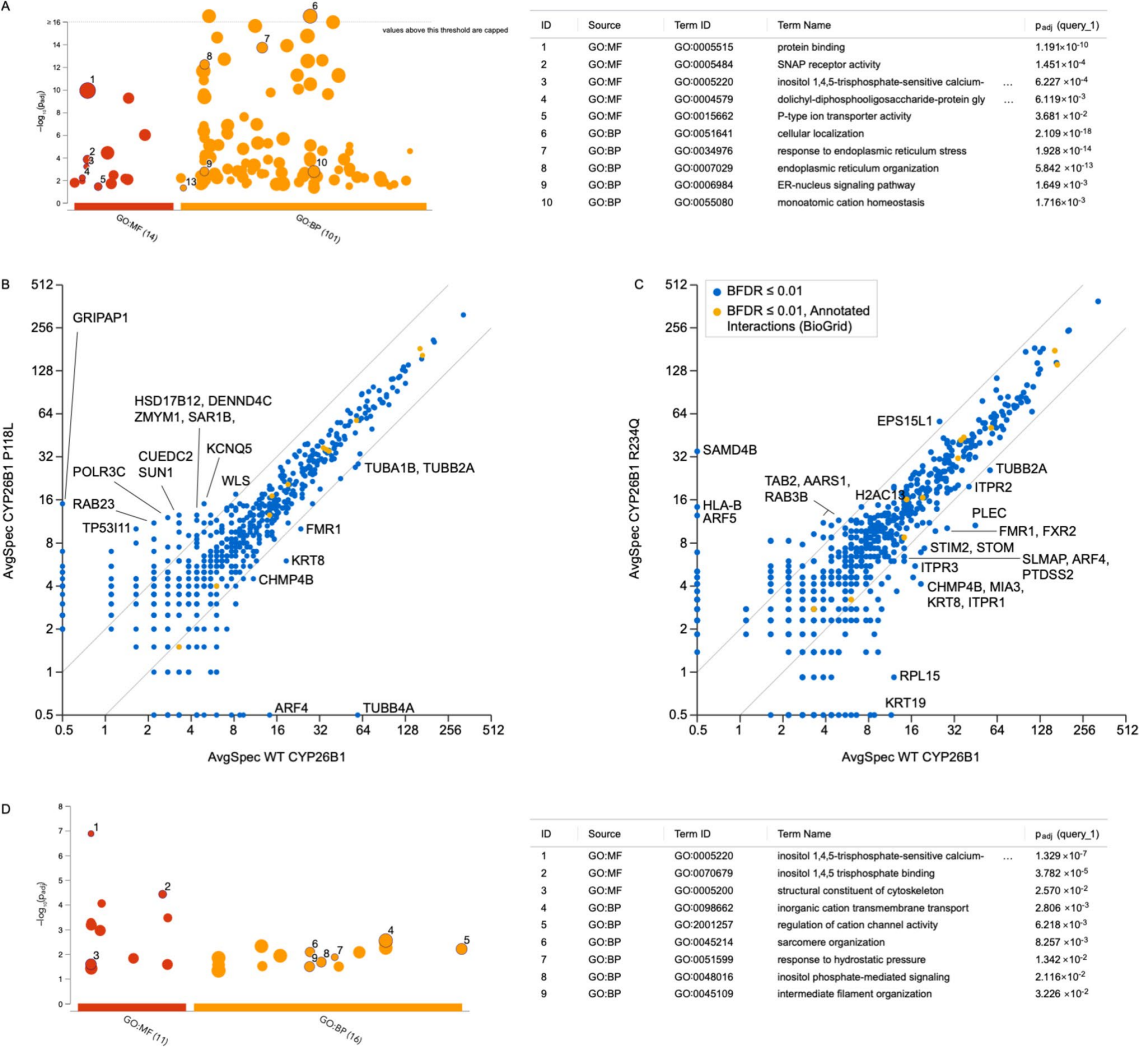
**A** CYP26B1 proteomic analysis reveals a loss of proximal associations with the p.Pro118Leu variant. Gene ontology analysis of the CYP26B1 proximal associations identified by BioID on the right. **B**, **C** Scatter plots comparing proximal associations with the wild-type (WT) and p.Pro118Leu (left) and p.Arg234Gln (right) CYP26B1 proteins. Labelled proteins had a Bayesian false discovery rate (BFDR) ≤ 1%, an average spectral count ≥ 10 in either the WT or mutant CYP26B1 BioID, and ≥ twofold change when comparing the two proteins. Grey lines denote the twofold change thresholds. **D** Gene ontology analysis of proteins whose proximal associations were altered with the p.Arg234Gln variant

BioID analysis confirmed previously reported associations with ANKLE2, ATF6, CDKAL1, CYP51A1, ESYT1, FKBP8, GPRC5C, OSBPL8, PDZD8, PKMYT1, and VRK2 proteins—most of which are overrepresented in ER gene ontology (GO) terms (Huttlin et al. 2021). A GO analysis of the 241 CYP26B1 BioID associations also reveals an overrepresentation of biological processes involving ER organization and signalling and homeostasis (Fig. 4A). While not part of driver GO terms, 13 GO terms were related to catabolic processes.

The proteome of WT CYP26B1 and the p.(Pro118Leu) variant was nearly identical, with only a few differences (mostly gained protein associations, Fig. 4B). However, only two GO terms relating to membrane proteins were deemed statistically significant. The p.(Arg234Gln) proteome was also very similar to that of the WT protein, but the variant weakened a greater number of CYP26B1 protein association (Fig. 4C). GO terms linked to the affected CYP26B1 associating proteins included inositol triphosphate calcium release, translation, and regulation of cation channels (Fig. 4D).

### Immunofluorescence

**Fig. 5:**
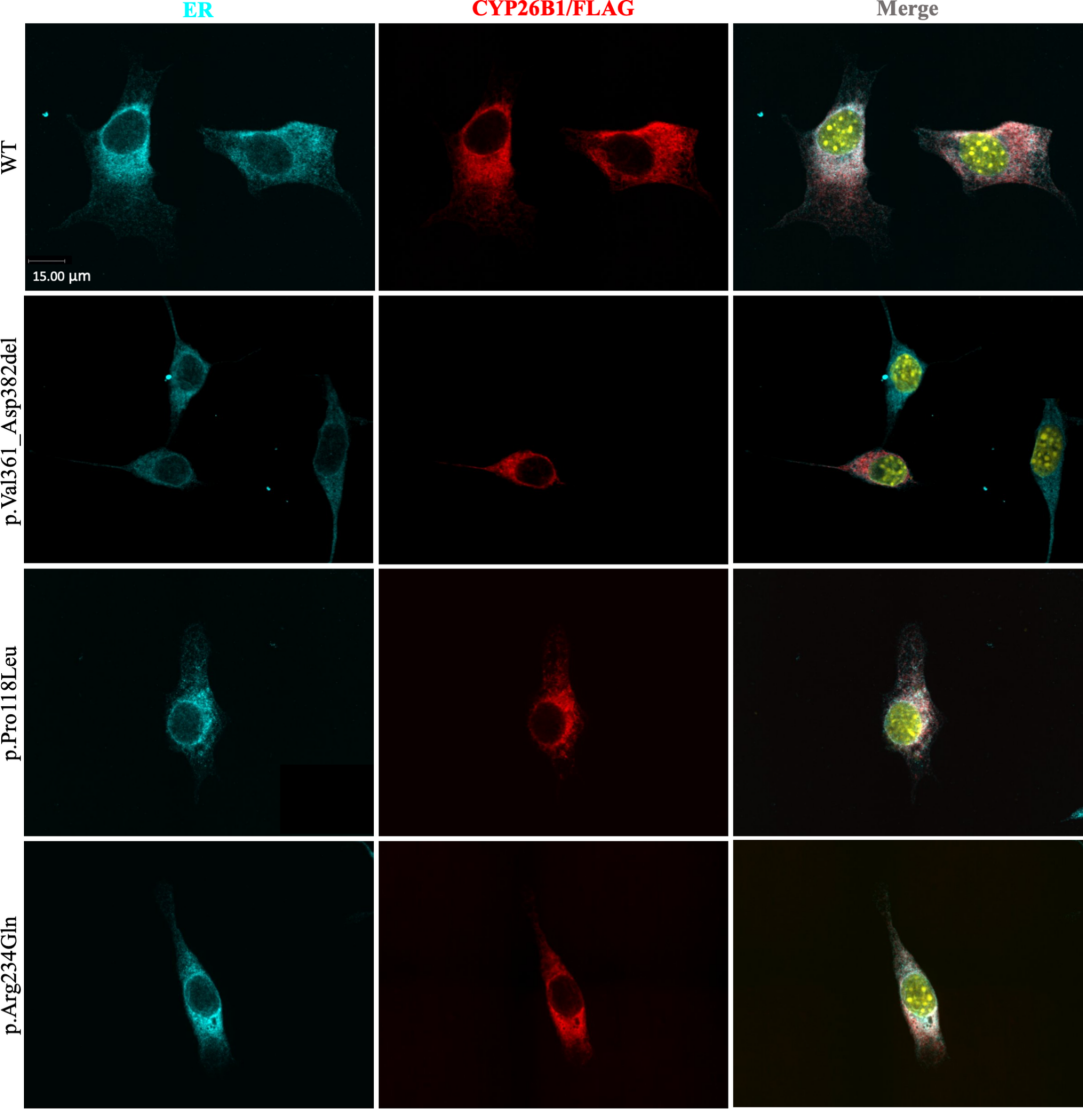
CYP26B1/FLAG proteins are properly localized. Representative images of immunofluorescence performed on NIH/3T3 cell line transiently transfected with wild-type (WT), p.Val361_Asp382del, p.Pro118Leu, and p.Arg234Gln CYP26B1/FLAG constructs (red). Calnexin (cyan) was used as an ER membrane marker and Hoechst was used for nucleus staining (here shown in yellow). Scale bar = 15 µm. *N* = 3 independent experiments

When examining the cellular localization of CYP26B1 plasmids alongside an ER membrane marker (calnexin), all constructs exhibited localization to the ER membrane (Fig. 5). The CYP26B1 protein signals appeared granulated with some foci formation in all constructs, with an increased loci formation in the mutants compared to the WT (online supplemental Fig. S6A, B). The expression of unfolded protein response (UPR) genes (*BiP, spliced Xbp1, Atf4, Atf6, Ern1, Perk*) were tested through quantitative PCR and results showed normal expression relative to the WT (online supplemental Fig. S6C). UPR expression and foci formation (*ERN1, BiP*, *spliced XBP1)* were also verified in stably transfected HEK293T/17 (online supplemental Fig. S7A, B) and presented similar results found in NIH/3T3 cells. It was assumed this foci formation represents only an artifact of the technique, possibly due to overexpression and/or aggregation caused by the affinity between CYP26B1 and the 3XFLAG tag*.*

## Discussion

Thus far, three homozygous and two compound heterozygous *CYP26B1* missense variants have been described in individuals displaying a similar phenotype (Fig. 3A) (Laue et al. 2011; Morton et al. 2016; Grand et al. 2021). Using an in-vitro assay, the p.(Arg363Leu) variant which causes a lethal phenotype displayed null enzymatic activity (Laue et al. 2011). In contrast, the p.(Ser146Pro) variant which causes a severe but survivable phenotype demonstrated reduced enzymatic activity (< 30%) (Laue et al. 2011). Enzymatic activity was not verified for a third homozygous variant (p.(Gly435Ser)) causing a mild skeletal phenotype (Morton et al. 2016). This variant was classified through in silico protein modelling. Protein modelling was also used to classify compound heterozygous missense variants (p.[(Arg363His)];[(Arg397Gln)], p.[(Arg234Gln);[(Gly126Cys)]) found in individuals with mild to intermediate skeletal malformations (Grand et al. 2021). Of interest, a further individual has been reported with compound heterozygous missense variants in *CYP26B1* (p.[(Glu177Lys)];[(His175Gln)]) and biallelic variations in the *NAGLU.* Although the phenotype (epilepsy, early onset epileptic encephalopathy, hypermyotonia, skull deformity, dilatation of the lateral ventricles and premature closure of fontanel) was different from the previous cases, it was attributed to the *CYP26B1* variants despite the p.(Glu177Lys) variant being previously classified as likely pathogenic and an absence of functional data for both variants (Li et al. 2018).

*CYP26B1* haploinsufficiency has also been reported with an abnormal phenotype. Microdeletions affecting *CYP26B1* and *EXOC6B* were identified in two individuals with brachycephaly, facial asymmetry, abnormal ears, mild joint contractures (elbow/knee) and neurological features (autism spectrum disorder, hyperactivity, and aggressive behaviour) (Wen et al. 2013). Although the authors demonstrated diminished *CYP26B1* expression compared to a control, a monoallelic deletion of *CYP26B1* has also been described in a healthy individual (Database of Genomic Variants/DGV, nsv953147) (Dogan et al. 2014). Thus, the larger microdeletion containing *CYP26B1* and *EXOC6B* may be the explanation behind the observed birth defects. More recently, a heterozygous stop-gain *CYP26B1* variant (c.86 C > A, p.(Ser29Ter)) was described in three family members (daughter, father, and grandfather), but an abnormal phenotype was only seen in the daughter and her father (Sarma et al. 2023). The possibility of a second pathogenic variant in this family was not completely ruled out, especially since there was consanguinity (father and mother first-degree cousins) suggesting an autosomal recessive inheritance pattern.

Our two unrelated families presented phenotypes within the *CYP26B1* spectrum. The siblings in family 1 (Fig. 1) showed an abnormal head shape without craniosynostosis of calvarial sutures, protruding eyes, low set ears, hearing loss, small mouth, impaired/decreased joint mobility, arachnodactyly, and learning disabilities. Apart from the absence of craniosynostosis, these features are also shared by other described individuals (Morton et al. 2016; Grand et al. 2021). The sibling’s variants were identified *in trans* (p.[(Pro118Leu)];[(Arg234Gln)]) and initially classified as VUS (PP1, PM2, PP3). We found that the p.(Pro118Leu) and p.(Arg234Gln) variants result in a partial loss in CYP26B1 enzyme activity (1.7× [*p* = 0.0019] and 2.3× [*p* = 0.0771] decrease, respectively) (Fig. 3E). Although the enzyme activity of the p.(Arg234Gln) variant data did not significantly differ from WT and other studied variants, it had been previously reported in another family with a mild/moderate CYP26B1 phenotype (Grand et al. 2021).

The fetus in family 2 (Fig. 2) demonstrated extensive spina bifida occulta, absence of clavicles, and femoral hypoplasia, findings not previously described as part of the CYP26B1-related phenotype. The variant (c.1083C > A) in the fetus was initially classified as synonymous (p.(Val361 =)), but its rarity (absent in all populational databases) and phenotypic similarities drove us to perform *in-silico* analysis which revealed a new cryptic donor splicing site. The cryptic splice site outcompetes the canonical splice sites, generating aberrant mRNA splicing (r.1081_1146del) (Fig. 2G, H) causing an in-frame deletion of 22 amino acids. DRL assay demonstrated that the shortened protein has a reduced ability to metabolize exogenous RA (almost 3.5-fold decrease compared to the WT) (Fig. 3E). In fact, the variant found in family 2 results in a more profound effect on enzyme function when compared to the previously reported p.(Arg363Leu) variant (*p* = 0.0063, Fig. 3E) (Laue et al. 2011). Thus, the notion of p.(Arg363Leu) leading to an absolute loss of function might need re-evaluation, given the current findings. If this supposition holds true, and a genetic alteration causing a complete loss of function has not been identified thus far, it is conceivable that such a loss could result in the termination of embryonic development in humans. This would explain the absence of instances, where both gene copies show premature termination and frameshift mutations causing the degradation of mRNA through nonsense-mediated decay. This hypothesis is supported by the fact that the variants identified here still yield intact RNA and are localized to the endoplasmic reticulum. Nonetheless, further investigations are imperative to establish the specific threshold of CYP26B1 enzyme activity required for the manifestation of either a severe or a mild phenotype.

To explore other mechanisms by which the missense variants in family 1 cause disease, we used BioID to compare protein–protein interactions (Fig. 4). Despite the limited characterization of the CYP26B1 proteome, the curated BioGrid database records 35 instances of protein interactions, all of which have been discovered through a solitary high-throughput experiment up to this point (Oughtred et al. 2021). BioID has the advantage of identifying direct and non-direct proximal associations and our analysis revealed 241 CYP26B1 proximal associations (online supplemental Table S4). A large number of proximal associations were also reported for other ER proteins, which was not particularly surprising given the number of proteins that transit through to the ER (Go et al. 2021). Currently, the only established functional protein interaction with CYP26B1 is the cytochrome p450 oxidoreductase (POR), another ER membrane protein that interacts with CYPs to recharge heme to a reduced state (Roberts 2020). Our data revealed that the interactome of the missense variants was nearly identical to the WT except for a few significant associations with proteins related to protein membranes for the p.(Pro118Leu) variant (Fig. 4A), and inositol triphosphate calcium release, translation, and regulation of cation channels proteins for the p.(Arg234Gln) variant (Fig. 4D). However, in both cases, a significant overrepresentation of proximal associations to ER proteins was also noted, which is expected since CYP26B1 is localized to the cytosolic side of the ER membrane (Go et al. 2021). Our data suggest that in addition to its enzymatic activity, CYP26B1 could be interacting with previously unassociated proteins involved with other biological events in the ER. Of note, further studies are necessary to validate the findings reported here. Nevertheless, the proteomic analysis allowed us to compare associations between the WT and mutant proteins.

Protein modelling (Fig. 3B, C) was also performed to explain how the pathogenic variants interfere with the folding and possibly the localization of CYP26B1. The phenotypic similarities with the proposita from family 2 and the previously reported individual could be due to disruption of the K-helix ExxR motif responsible for heme stabilization (Fig. 3D, online supplemental Fig. S5) (Laue et al. 2011). The p.Val361_Asp382del pathogenic variant removes the ExxR motif and substrate recognition site 5, related to the stabilization of the meander loop and the maintenance of the CYP tertiary structure and the orientation of the substrate near the heme center, respectively (Hasemann et al. 1995; Seifert and Pleiss 2009; Sirim et al. 2010). Therefore, we suspected the p.Val361_Asp382del variant disrupts protein folding and localization. However, our localization assay showed proper co-localization with the ER marker indicating that despite the loss of an important domain, the protein remained properly localized (Fig. 5).

The skeletal defects observed in the *CYP26B1*-related conditions are caused by the excess of RA (Laue et al. 2011; Lind et al. 2017, 2018). Overabundance of RA is known to increase chondrocyte proliferation and maturation, and to accelerate the transition of osteoblasts to osteocytes (Laue et al. 2011; Nilsson et al. 2016). In a normal growth plate, chondrocytes are arranged in a columnar pattern with clearly demarcated zones (resting zone, proliferative, and hypertrophic zones) (Kronenberg 2003). H&E staining of the fetal growth plate from family 2shows generalized disorganization with resting zone chondrocyte hypercellularity and a short proliferative and hypertrophic zone (Fig. 2E). In addition, the proliferative zone cells were small with less cytoplasm, and the hypertrophic zone cells were few and more widely spaced than expected. These histological findings are comparable with data from hypervitaminosis A in rats, *Cyp26b1 knockout* mice, and zebrafish. All studies show disrupted cartilage and bone homeostasis with increased osteoblast and osteoclast activity, accelerated mineralization with the premature formation of osteocytic cells, and reduced chondrocyte proliferation (Laue et al. 2008, 2011; Spoorendonk et al. 2008; Minegishi et al. 2014; Shen et al. 2022).

Since *CYB26A1* but not *CYP26B1* is known to be expressed in somites forming the human spine, we are puzzled by the extensive spina bifida occulta observed in the fetus from family 2. Nonetheless, *Cyp26b1* exhibits expression in zebrafish during the somitogenesis phase, and a zebrafish *knockdown* model has documented instances of excessive vertebral column ossification (Hernandez et al. 2007; Spoorendonk et al. 2008). Interestingly, *Cyp26b1* is also expressed at specific levels of the differentiating murine upper and lower thoracic spinal cord but spinal defects were not reported in the *knockout* mouse model (Abu-Abed et al. 2002; Yashiro et al. 2004). However, hypervitaminosis A causing RA accumulation in mouse embryos results in spinal defects (Alles and Sulik 1990; Sakai et al. 2001). It is possible that the p.Val361_Asp382del variant leads to RA accumulation that perfuses nearby tissues (from limb bud to somites) at the early stages of development overwhelming the local CYP (*CYP26A1*) present in the somites. The buildup of RA thus causes the spinal defects (Sakai et al. 2001; Yashiro et al. 2004; White et al. 2007; Cunningham and Duester 2015).

**Fig. 6:**
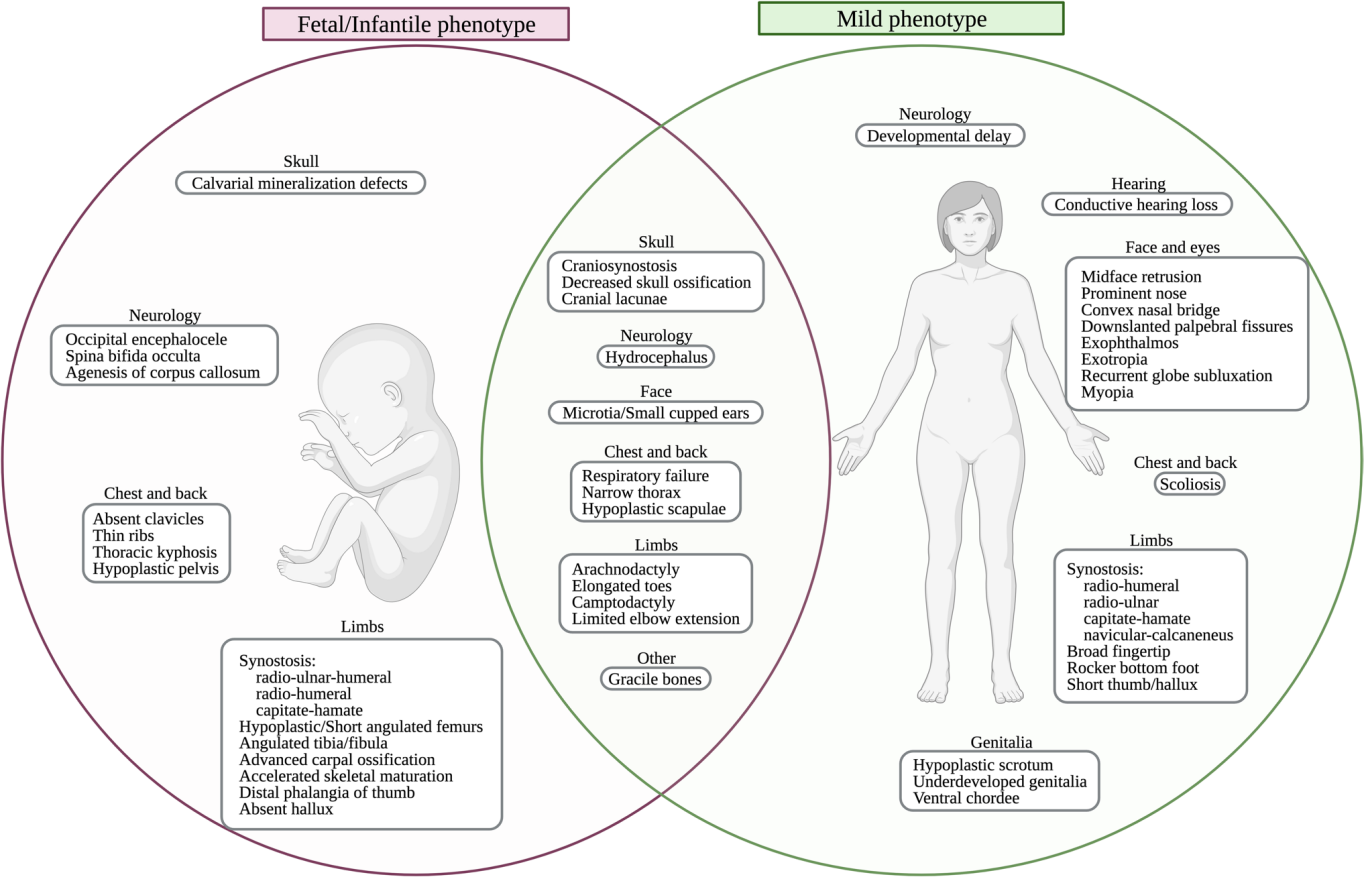
Main clinical and skeletal features caused by pathogenic variants in *CYP26B1*. The figure shows the features that are present in the lethal/infantile phenotype (in red), mild phenotype (in green) and shared features in the middle. All terms used in the figure are shown with their respective HPO term in the online supplemental Table S5

Only a few pathogenic variants in the *CYP26B1* gene have been published thus making genotype–phenotype correlations difficult. Our review of all reported biallelic *CYP26B1* variants published up to now (Laue et al. 2011; Morton et al. 2016; Li et al. 2018; Grand et al. 2021), including the families reported here, indicate the following cardinal features of the genotype–phenotype spectrum: (1) higher retained enzymatic activity is characterized by craniosynostosis, ear malformations with hearing loss, and intellectual disability and (2) lower enzymatic activity is typified by neural tube defects (encephalocele, spina bifida), decreased mineralization (calvarium defects, clavicular absence, femoral hypoplasia and oligodactyly (Fig. 6).

In summary, we have successfully detected a novel splicing variant of *CYP26B1* that results in a protein deletion (p.Val361_Asp382del). We also show that the degree of retained enzymatic activity directly drives the severity of the phenotype. Our findings contribute to the expanding range of genotypic and phenotypic anomalies associated with disruptions in retinoic acid catabolism, as evidenced by the cases presented in these two families.

### Supplementary Information

Below is the link to the electronic supplementary material.

Supplementary file1 (PDF 10784 KB)

Supplementary file2 (XLSX 444 KB)

## Data Availability

Mass spectrometry data for the BioID has been deposited to the MassIVE repository (https://massive.ucsd.edu/ProteoSAFe/static/massive.jsp) and assigned the accession number MSV000091800 (ftp://massive.ucsd.edu/MSV000091800). The ProteomeXchange accession is PXD041830.
