# Supplementary material for: CYP26B1-related disorder: expanding the ends of the spectrum through clinical and molecular evidence
Source: Hum Genet. 2023 Sep 27;142(11):1571–86. doi: 10.1007/s00439-023-02598-2 (PMC10602971; doi:10.1007/s00439-023-02598-2)
Supplement: Supplementary file 1 — Supplementary file1 (PDF 10784 KB) [file 439_2023_2598_MOESM1_ESM.pdf]

SUPPLEMENTARY INFORMATION

Title: CYP26B1-related disorder: expanding the ends of the spectrum through clinical and molecular evidence

Human Genetics

Authors: Karina C. Silveira[1], Inara Chacon Fonseca[2], Connor Oborn[1], Parker Wengryn[1], Saima Ghafoor[1], Alexander Beke[1], Ema S. Dreseris[3], Cassandra Wong[4], Aline Iacovone[5], Carrie-Lynn Soltys[1], Riyana Babul-Hirji[6], Osvaldo Artigalas[7], Arthur Antolini[8], Anne-Claude Gingras[4,9], Eric Campos[3,9,†], Denise P. Cavalcanti[5, †,*], Peter Kannu[1, †,*]

Corresponding authors:

Peter Kannu, Department of Medical Genetics, University of Alberta, Edmonton, Alberta, T6G 2H7, Canada. Tel: +1 (780) 492 9044; Fax: (780) 492 1998; E-mail: kannu@ualberta.ca

Denise P. Cavalcanti, Grupo de Displasias Esqueléticas, Genética Médica, FCM, UNICAMP, R. Tessália V de Camargo, 126, 13083-887, Campinas, SP, Brazil. Tel: +55 (19) 3521 0385. Email: denisepcavalcanti@gmail.com; denisepc@unicamp.br

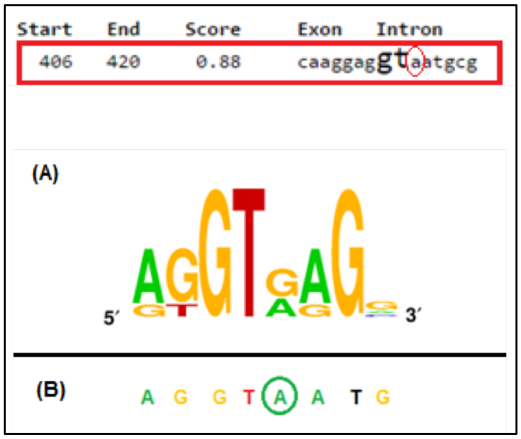

Fig S1: *CYP26B1* donor splice site consensus. The c.1083C>A variant changes the consensus region creating an alternative splice site right before it (circle). A) Consensus region. B) new sequence with the variant c.1083C>A in exon 5.

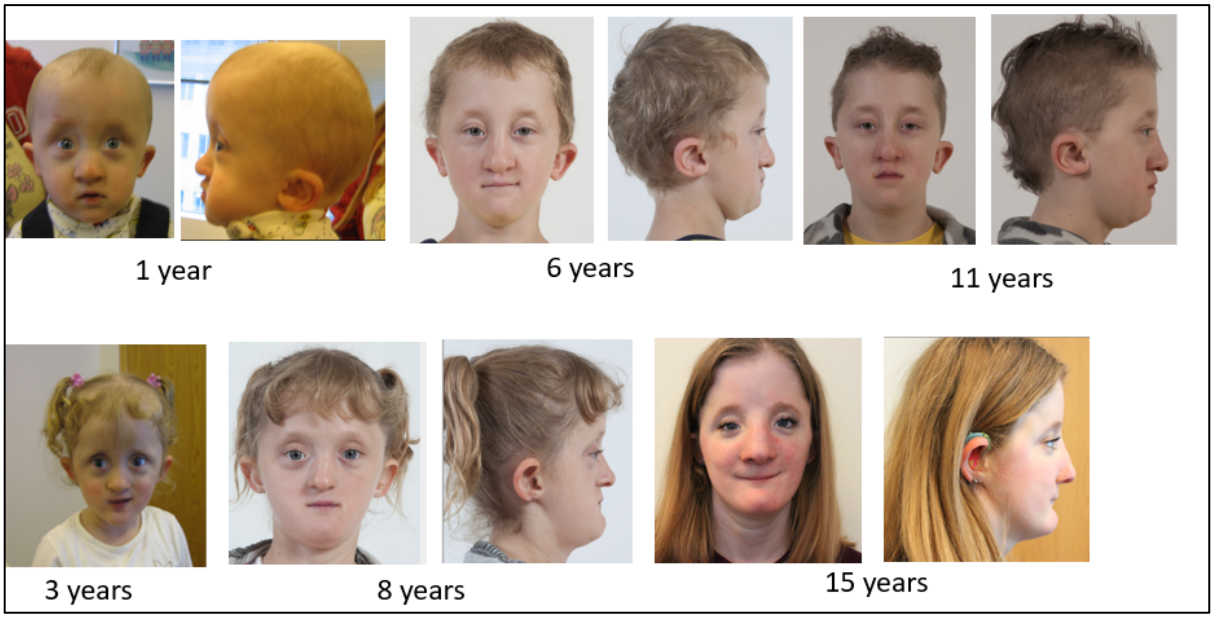

Fig S2: Family 1 follow-up.

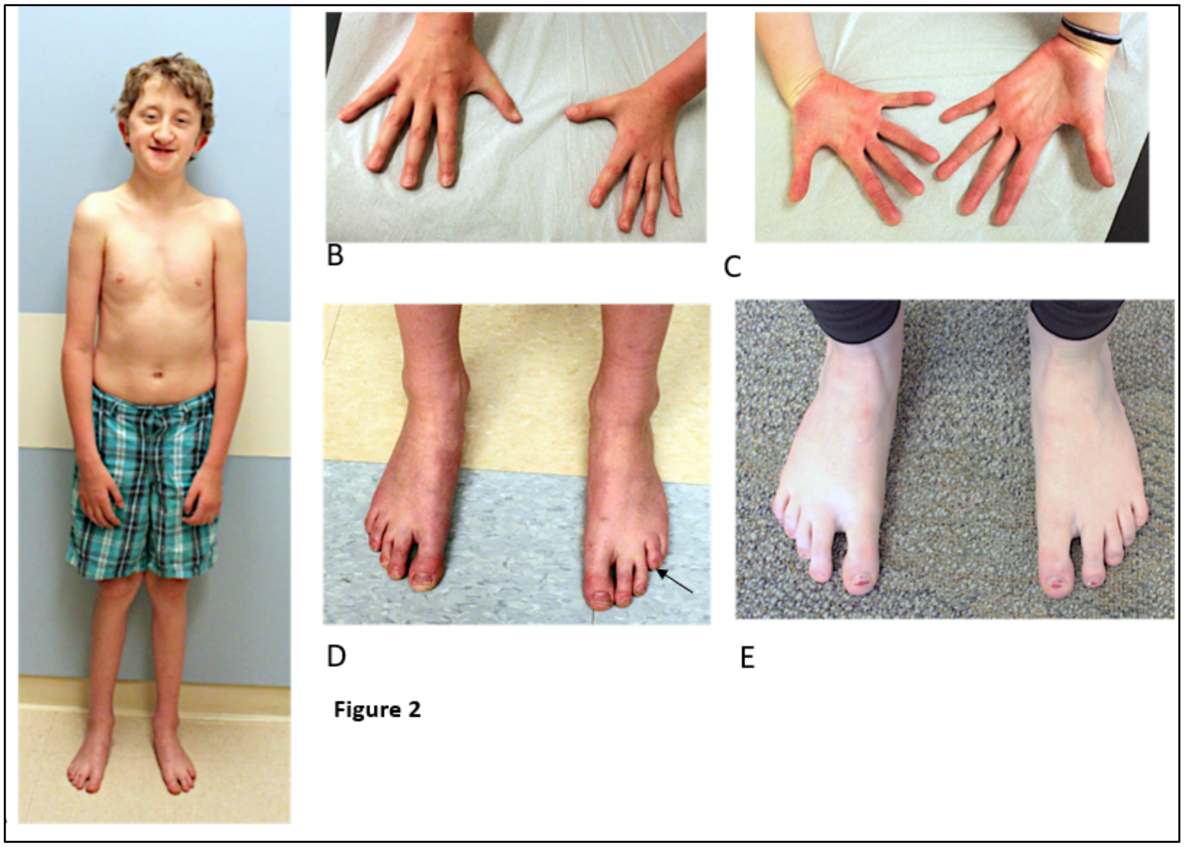

Figure S3: Feet and hands from individuals from family 1.

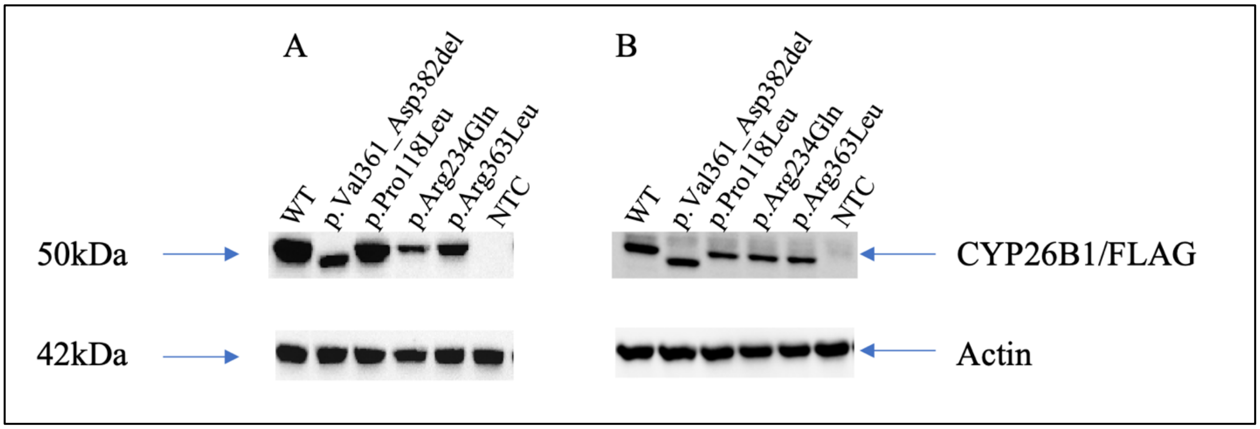

Fig S4: Expression vector protein verified by western blot in HEK293T/17 (A) and NIH/3T3 (B).

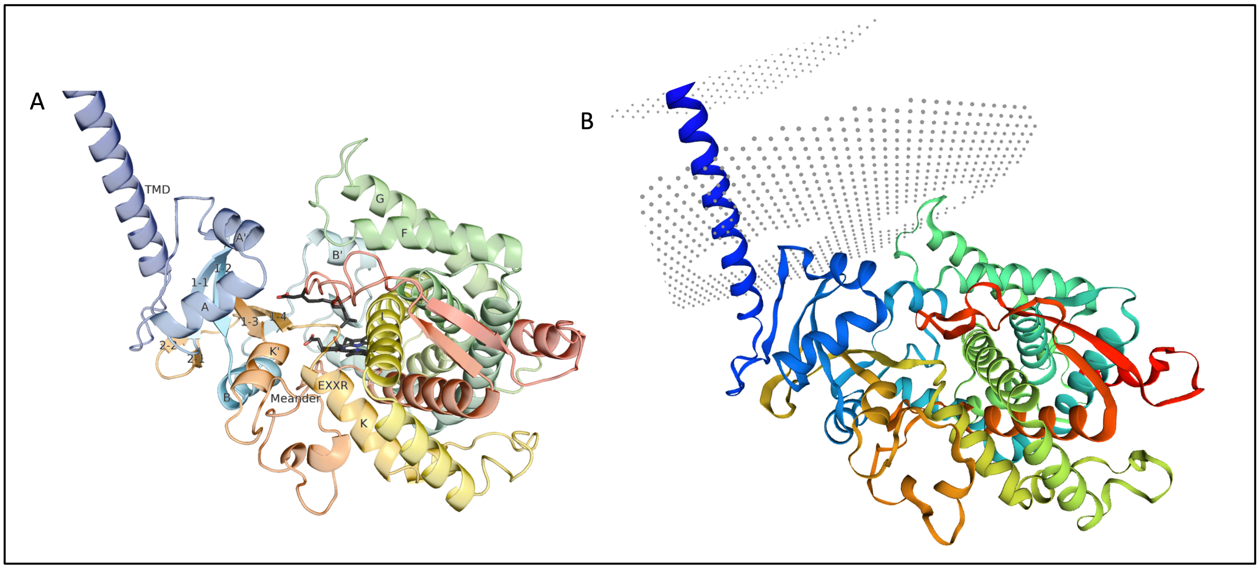

Fig S5: A) CYP26B1 protein structural overview. The 3D model of wildtype CYP26B1 chromatically labeled from N terminus (blue) to C terminus (red), helices and strands are labeled as per P450 structural conservation as letters and pairs of numbers respectively. Retinoic acid is positioned above the heme in the core and are both labeled black. **B) CYP26B1 relative to membranes.** Predicted wildtype structure aligned with a membrane annotation identified 3 points of contact on the CYP26B1 architecture. Labelled chromatically from N terminus (blue) to C terminus (red).

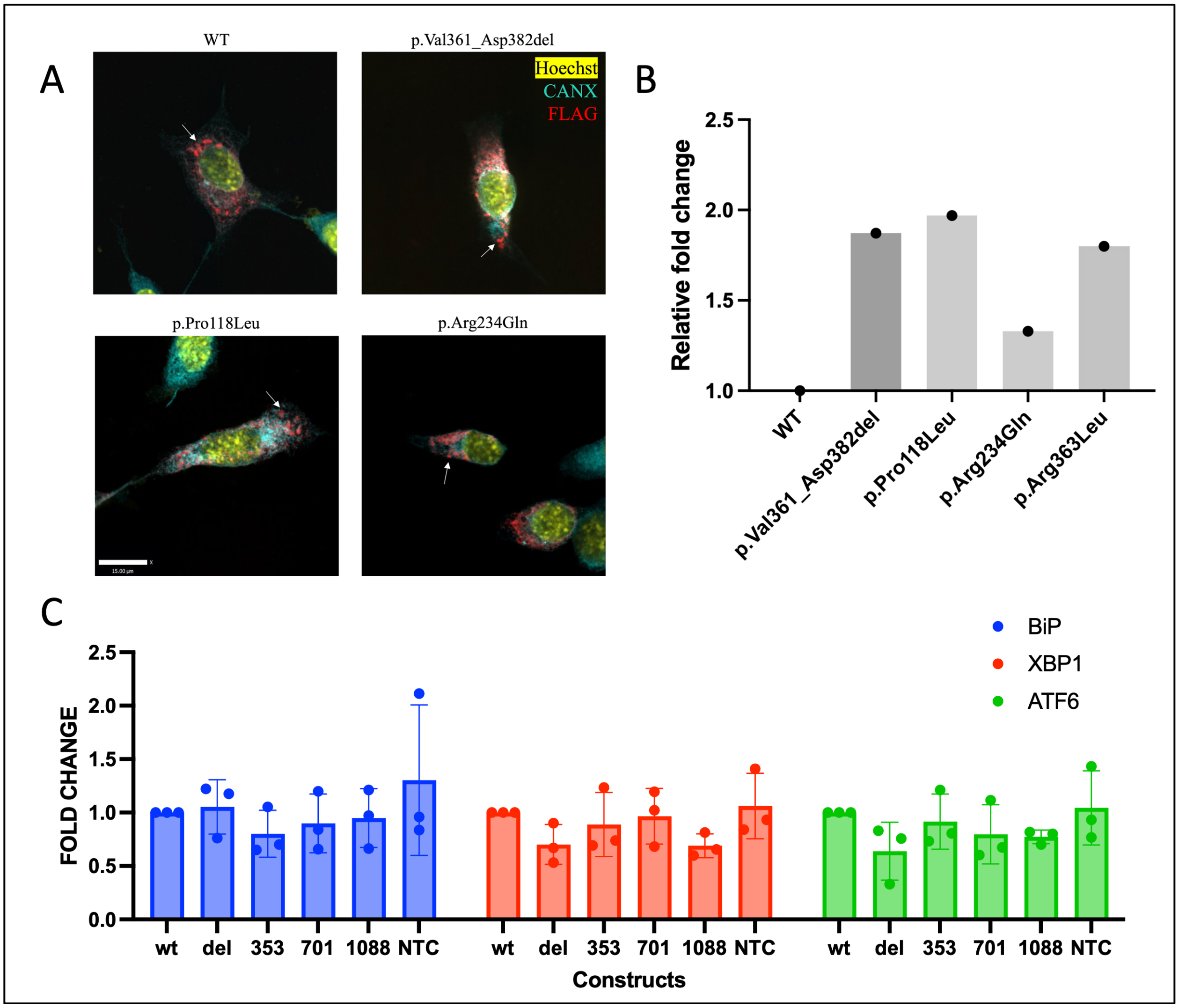

Fig S6: A) CYP26B1 foci formation (white arrow) in transiently transfected NIH3T3 cells. B) Counting of transiently transfected NIH/3T3 cells presenting the foci formation relative to the WT. At least 166 cells were verified for each construct. C) UPR genes (*BiP, XBP1, ATF6*) expression verified in transiently transfected NIH/3T3 cells by qPCR.

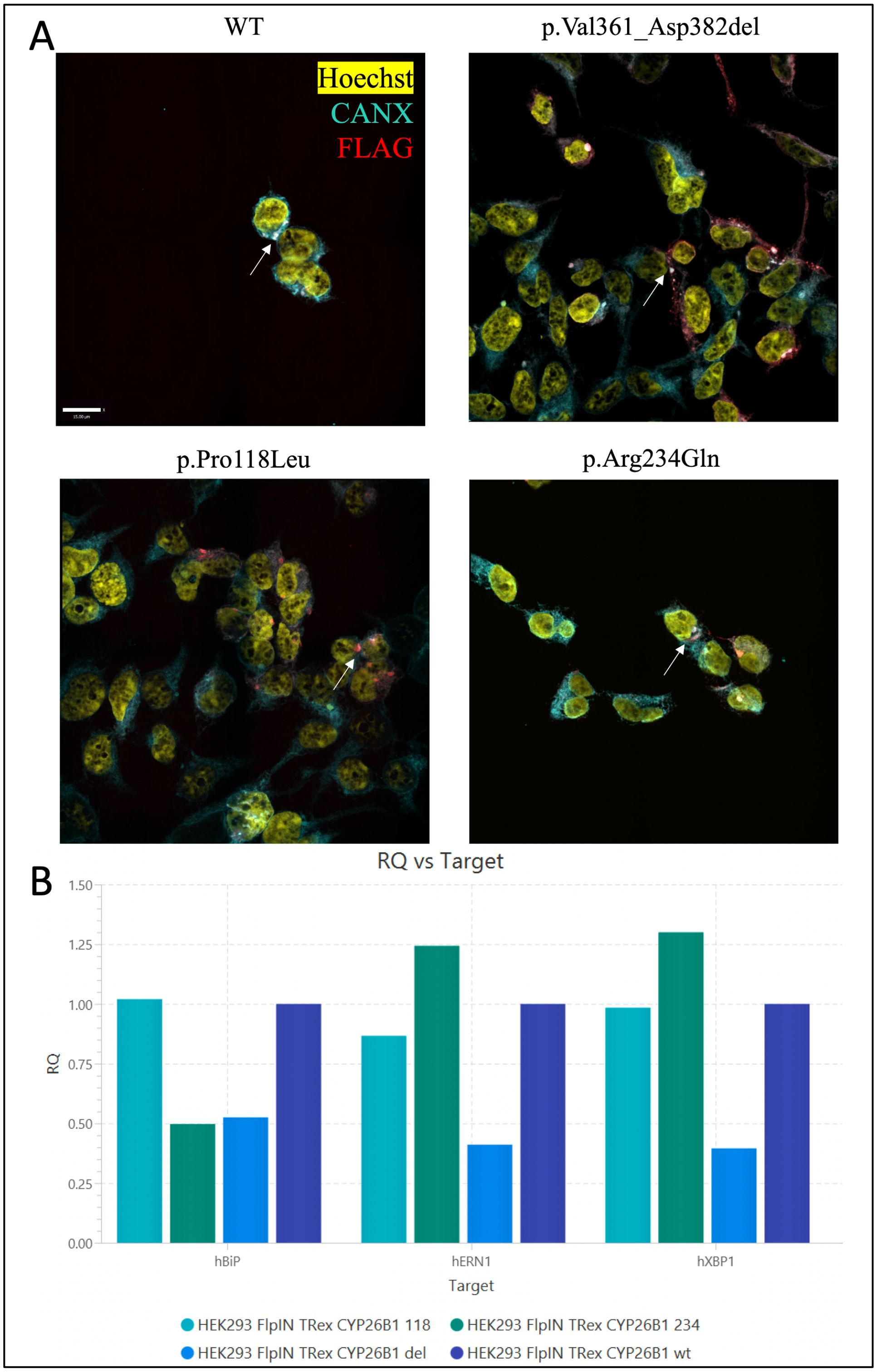

Fig S7: A) CYP26B1 foci formation (white arrow) in stably transfected HEK293 cells. B) UPR genes (*BiP, ERN1, XBP1*) expression verified in stably transfected HEK293 cells by qPCR.

Table S1: Primer sequences.

Name	Primer sequence (5’-3’)
***CYP26B1* primers – PCR/Sanger sequencing**	
CYP26B1.E1F	gcccctaagcccgctataaag
CYP26B1.E1R	gacccaggcaagtccatcttg
CYP26B1.E1IntF	AGGGCTTGGATCTGGTGTCGGC
CYP26B1.E2F	cagttcttgtgcacgctatgaa
CYP26B1.E2R	agtcttcaagcatggtgtgcaa
CYP26B1.E3F	ccaaagacttgctggcagctg
CYP26B1.E3R	ctttgtccatgtgccctgtgc
CYP26B1.E4F	gcacagggcacatggacaaag
CYP26B1.E4R	agctcagaatggagcctgggt
CYP26B1.E5F	gctcagaggaagcagaattcac
CYP26B1.E5R	tgaagtccctgaaatggaggct
CYP26B1.E6F	aaggcaggtcttgaacatctgg
CYP26B1.E6R	TCCCACACACAGGTTTCTACCT
**MUTAGENESIS – Expression plasmid**	
CYP.cDNA.g1088tR	tgggcgtgaacaggagcatgacctccttg
CYP.cDNA.c353t.R	cgggtgctgcgaagccactcggtgc
CYP.cDNA.g701a.F	cttcagtggctaccagcggggcattcagg
CYP.cDNA.g701a.R	cctgaatgccccgctggtagccactgaag
CYP.cDNA.c353t.F	gcaccgagtggcttcgcagcacccg
CYP.cDNA.g1088tF	caaggaggtcatgctcctgttcacgccca
CYP26B1.delcDNAF	tggggatctggaaaccctccttgatgacgcag
CYP26B1.delcDNAR	ctgcgtcatcaaggagggtttccagatcccca
**MINIGENE ASSAY – genomic DNA**	
CYP26B1.Intron4.F	GCTCAGAGGAAGCAGAATTCAC
CYP26B1.Intron5.R	TCCCACACACAGGTTTCTACCT
**MINIGENE ASSAY – mutagenesis**	
CYP26B1.delCDNA.1083C>A.F	CGTCATCAAGGAGGTAATGCGCCTGTTCACG
CYP26B1.delCDNA.1083C>A.R	CGTGAACAGGCGCATTACCTCCTTGATGACG
**MINIGENE ASSAY – transcript amplification**	
cDNA specific primer (pET01 vector)	GATCCACGATGC
PCR. CYP26B1.F	ACTGTGCTGGAGAAGCTGCG
PCR.CYP26B1.R	GGTCGAACACGTTCACGTCTT

Table S2: Physical characteristics of our patients from family 1 compared with a previously described individual with biallelic variants in the *CYP26B1* gene who has survived infancy.

	**Proband Family 1**	**Sister Family 1**	**Morton *et al.*** (1)
Height, percentile	75	95	50
Weight, percentile	25-50	50	U
Head circumference, percentile	25-50	25	<3
Turribrachycephaly	+	+	+
Hypertelorism	-	+	+
Wide orbital fissures	+	+	U
prominent nose	+	+	+
Malar hypoplasia	+	+	+
bulbous nose tip	+	+	+
small mouth	+	+	+
protruding ears	+	+	+
low set ears	+	+	+
Conductive hearing loss	+	+	+
high palate arch	+	+	+
crowded teeth	+	+	U
Facial asymmetry	-	-	-
Pointy Chin	+	+	-
Craniosynostosis	-	-	+
Skull defect/Cranium bifidum	-	-	+
Abnormal spine curvature	-	+	+
Radio-ulnar limitation	+	+	-
Radio-humeral limitation	-	-	+
Pectus excavatum	+	-	-
Prominent pisiform bone	+	+	U
Advanced bone age	-	-	U
Short forth metatarsal	+	-	+
Arachnodactyly	+	+	+
Camptodactyly	-	-	+
Fusion metatarsal and cuneiform	+	+	U
Bone mineral density	N	N	N
Developmental delay	+	+	+
Learning disability/ID	+	+	+
Autism and behavioral issues	-	-	-

(+): present; (-): not present; U: unknown; N: normal; NA: not applicable

Table S3: Comparison of the clinical and radiological findings of the fetuses reported by Laue et al. (2011) and the fetus of family 2 presented in this study.

	Laue et al., 2011 (2) Family 1			Present study Family 2
	II.1	II.2	II.3	IV.1
Parental consanguinity				y
Gestational age (weeks)	36	15	12	33
Neural tube defects	+[a]	+[a]	+[a]	+[b]
Calvarial mineralization defects	+			+
Hydrocephalus				+
Agenesia of corpus callosum				+
Microtia				+
Narrow thorax	+			+
Short limbs	+	+	+	+
Limited elbow extension	+			+
Aracnodactyly appearance	+			+
Oligodactyly	+[c]			+[d]
Distal aphalangia of thumbs	+			+
Radio-ulnar-humeral fusion	+			+
Hypoplasia of scapulae	+			+
Advanced carpal maturation	+			+
Skeletal mineralization decreased				+
Absence of clavicles				+
Thin ribs				+
Torax kyphosis				+
Spina bifida occulta				+
Hypoplasia of pelvis	+			?
Angulated bones in lower limbs	+[e]	+[e]	+[e]	+[f]
Elongated toes	+			+
Absence of halux	+	+	+	+

[a] Encephalocele. [b] Generalized spina bifida occulta. [c] Only the hallux is absent. [d] Hallux and other fingers and toes are absent. [e] Angulated femur. [f] Angulated tibia.

Table S4: BioID results (Excel file)

Table S5: HPO terms described in figure 6.

Phenotype	HPO		
Absent hallux	HP:0012386		
Accelerated skeletal maturation	HP:0005616		
Advanced ossification of carpal bones	HP:0004233		
Agenesis of corpus callosum	HP:0001274		
Arachnodactyly	HP:0001166		
Broad fingertip	HP:0011300		
Calvarial mineralization defects			
Camptodactyly	HP:0012385		
Conductive hearing loss	HP:0000405		
Convex nasal bridge	HP:0000426		
Cranial lacunae			
Craniosynostosis	HP:0001363		
Cupped ear	HP:0000378		
Decreased skull ossification	HP:0004331		
Development delay	HP:0012758		
Distal phalangia of thumbs			
Down slanted palpebral fissures	HP:0000494		
Elongated toes	HP:0010511		
Exophthalmos	HP:0000520		
Exotropia	HP:0000577		
Exotropia	HP:0000577		
Gracile bones	HP:0003100		
Hydrocephalus	HP:0000238		
Hypoplastic pelvis	HP:0008839		
Hypoplastic scapulae	HP:0000882		
Hypoplastic scrotum	HP:0000046		
Limited elbow extension	HP:0001377		
Microtia	HP:0008551		
Midface retrusion	HP:0011800		
Myopia	HP:0000545		
Narrow thorax	HP:0000774		
Occipital encephalocele	HP:0002085		
Oligodactyly	HP:0012165		
Prominent nose	HP:0000448		
Recurrent globe subluxation			
Respiratory failure			
Rocker bottom foot	HP:0001838		
Scoliosis	HP:0002650		
Short angulated femur			
Short thumbs / hallux	HP:0009778	HP:0010109	
Spina bifida occulta	HP:0003298		
Synostosis	HP:0100240	HP:0004278	HP:0100238
Thin ribs	HP:0000883		
Thoracic kyphosis	HP:0002942		
Underdeveloped genitalia	HP:0000050	HP:0012815	
Ventral chordee	HP:0000041		

References

1. Morton, J.E.V., Frentz, S., Morgan, T., Sutherland-Smith, A.J. and Robertson, S.P. (2016) Biallelic mutations in CYP26B1: A differential diagnosis for Pfeiffer and Antley–Bixler syndromes. *Am. J. Med. Genet. Part A*, **170**, 2706–2710.
2. Laue, K., Pogoda, H.M., Daniel, P.B., Van Haeringen, A., Alanay, Y., Von Ameln, S., Rachwalski, M., Morgan, T., Gray, M.J., Breuning, M.H., *et al.* (2011) Craniosynostosis and multiple skeletal anomalies in humans and zebrafish result from a defect in the localized degradation of retinoic acid. *Am. J. Hum. Genet.*, **89**, 595–606.
